# In Vivo Effects of Neostigmine and Physostigmine on Neutrophil Functions and Evaluation of Acetylcholinesterase and Butyrylcholinesterase as Inflammatory Markers during Experimental Sepsis in Rats

**DOI:** 10.1155/2019/8274903

**Published:** 2019-01-20

**Authors:** Diane I. Bitzinger, Michael Gruber, Simon Tümmler, Manuela Malsy, Timo Seyfried, Florian Weber, Andreas Redel, Bernhard M. Graf, York A. Zausig

**Affiliations:** ^1^Department of Anesthesiology, University of Regensburg, Regensburg, Germany; ^2^Department of Pathology, University of Regensburg, Regensburg, Germany

## Abstract

**Introduction:**

Recent studies have shown that acetylcholinesterase (AChE) and butyrylcholinesterase (BChE) may serve as important diagnostic and therapeutic targets in sepsis. Since polymorphonuclear neutrophils (PMNs) play a pivotal role in the early phase of sepsis, we evaluated the potential therapeutic effects of cholinesterase inhibitors on PMN functions during cecal ligation and puncture- (CLP-) induced sepsis and investigated the roles of AChE and BChE as inflammatory markers under standardized experimental conditions.

**Methods:**

Sham surgery or CLP was performed in male Wistar rats (*n* = 60). Animals were randomized into four groups: physostigmine, 100 *μ*g/kg; neostigmine, 75 *μ*g/kg; 0.9% saline (control group); and sham group, each applied four times over 24 h. The levels of reactive oxygen species (ROS) production and CD11b/CD62l expression were quantified by flow cytometry at *t* = 0, 6, 15, 20, and 24 h. Blood gas analysis as well as AChE and BChE activity levels was measured by validated point-of-care measurements. Clinical scores and survival times were determined.

**Results:**

CLP induced a significant increase in ROS production and CD11b upregulation by rat PMNs. Treatment with physostigmine or neostigmine significantly reduced ROS production and CD11b upregulation by PMNs 20 h after CLP induction. In physostigmine-treated animals, survival times were significantly improved compared to the control animals, but not in neostigmine-treated animals. While AChE activity significantly decreased in the control animals at *t* > 6 h, AChE activity did not change in the sham group. BChE activity decreased at *t* > 20 h in the control animals.

**Conclusion:**

While AChE activity may serve as an acute inflammatory marker, BChE activity shows a delayed decrease. Administration of centrally acting physostigmine in CLP-induced sepsis in rats has protective effects on PMN functions and improves survival times, which may be of interest in clinical practice.

## 1. Introduction

Sepsis and septic shock are major challenges in modern intensive care. Treatment of the causative infection is fundamental for successful treatment of sepsis, but the course can be positively influenced by supportive and adjuvant measures. The body's first defense against invading pathogens or tissue injury is the innate immune system, which involves a complex network of cytokines produced by activated leukocytes. However, overproduction of these mediators and their release into the bloodstream are characteristics of the early phase of sepsis [1], resulting in secondary tissue injury, organ dysfunction, and systemic inflammation with potentially lethal multiorgan failure.

Polymorphonuclear neutrophils (PMNs) are multifunctional cells that play a pivotal role in the inflammatory injury of sepsis. PMN rolling and adherence on activated endothelium are critical steps in transendothelial migration. L-selectin (CD62l) is an adhesion molecule on the surface of PMNs that promotes rolling. Firm adherence and diapedesis are mediated by Mac-1 (CD11b/CD18). Furthermore, the generation of reactive oxygen species (ROS) by PMNs plays an essential role in PMN-mediated host defense [2, 3]. Further clarification of the role of PMNs and therapeutic manipulation of PMN-mediated actions in sepsis is imperative.

Previous publications have indicated that the activation of central cholinergic signaling and the central action of cholinesterase inhibitors activate the cholinergic anti-inflammatory pathway (CAP) and alleviate systemic inflammation in inflammatory bowel disease or murine endotoxemia [4–8]. Peter et al. showed in a model of experimental endotoxemia that the number of rolling leukocytes was significantly reduced by the application of physostigmine, a cholinesterase inhibitor which crosses the blood-brain barrier [9]. Hofer and colleagues demonstrated that physostigmine as well as neostigmine, a cholinesterase inhibitor which only acts in the periphery, improved survival in a murine cecal ligation and puncture (CLP) model [10]. These data are quite different from those of the previous studies by Akinci et al., which failed to demonstrate protective effects of neostigmine in a murine model of endotoxin-induced septic shock [11]. In addition, Kox et al. reported no protective effect of neostigmine on ventilation-induced cytokine responses, lung injury, and function [12]. The cause for the variation in results between these studies with centrally acting physostigmine and peripherally acting neostigmine is still in focus of current research.

Recent studies have shown that acetylcholinesterase (AChE) and butyrylcholinesterase (BChE) serve as diagnostic markers of low-grade systemic inflammation [13–15]. Rapid changes in cholinesterase activity have also been reported in patients after acute trauma, infections, burns, and critical illness [16–20]. Both enzymes may serve as indicators of systemic inflammation and have a remarkable predictive value for mortality in critically ill patients. However, due to high variability in the onset, etiology, and progress of clinical conditions among patients, determining whether changes in the enzyme activity are correlated with the emergence of disease or are affected by other concomitant factors is difficult.

Since the role of AChE and BChE activity has not yet been evaluated under standardized experimental conditions, we investigated the changes in enzyme activity during CLP-induced inflammatory responses. In this comparative study, we further investigated the effects of peripherally acting neostigmine and centrally acting physostigmine on typical immune functions of PMNs, such as adhesion and ROS generation, and examined the potential of cholinesterase inhibitors for use in the early treatment of sepsis.

## 2. Materials and Methods

### 2.1. Animals

The animal experiments were conducted in accordance with the German laws regulating animal care, the European Communities Council Directive (2010/63/EU), and institutional guidelines (Zentrale Tierlaboratorien, Universität Regensburg). All animal experiments were approved by the Zentrale Tierlaboratorien, Universität Regensburg, and the local ethics committee (Regierung der Oberpfalz, Regensburg, Germany) under permit number 54-2532.1-21/12. Rats were housed in a temperature-controlled room at 22 ± 0.5°C. Free access to food and water was allowed throughout the experimental period. In total, 60 male Wistar rats (Charles River, Sulzfeld, Germany) weighing 250-350 g each were included in the study. All efforts were made to prevent animal suffering.

### 2.2. Study Design

**Figure 1:**
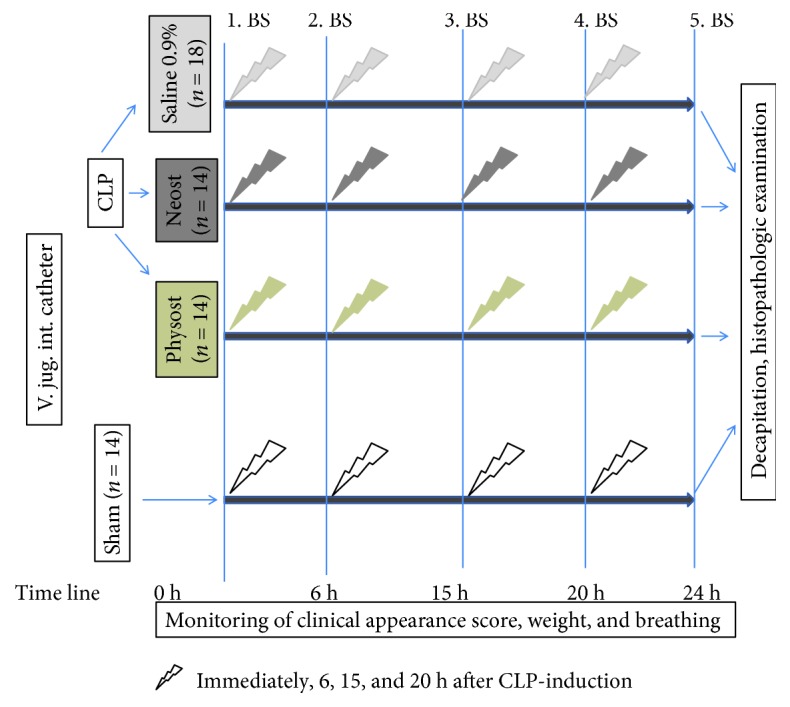
Illustration of the experimental proceeding. Animals were randomized into four groups: the sham, physostigmine, neostigmine, and control groups. Neostigmine (75 *μ*g/kg), physostigmine (100 *μ*g/kg), and 0.9% saline (=control) were administered immediately, 6, 15, and 20 h after CLP induction. Blood samples (BS) were drawn immediately, 6, 15, 20, and 24 h after CLP/sham procedures. The animals were evaluated regularly (clinical appearance score, breathing rate). All surviving animals were euthanized after 24 h by decapitation, and their organs were isolated for histopathologic examination.

Figure 1 describes the experimental protocol. A total of 60 rats were randomly allocated to 4 groups: (i) the control group, *n* = 18; (ii) the physostigmine group, *n* = 14; (iii) the neostigmine group, *n* = 14; and (iv) the sham group, *n* = 14. In detail, during a 20 to 25 min time period, a catheter was implanted in the vena jugularis interna and CLP or sham operation procedures were performed under anesthesia. At the end of surgery, physostigmine (100 *μ*g/kg body weight; Dr. F. Köhler Chemie, Bensheim, Germany), neostigmine (75 *μ*g/kg body weight; Rotexmedica, Trittau, Germany), or 0.9% saline was immediately administered by intraperitoneal injection. The saline group served as the control group. These animals received only saline 0.9% in equivalent volumes. Administration of physostigmine, neostigmine, or saline 0.9% was repeated after 6, 15, and 20 h. Blood samples were drawn immediately, 6, 15, 20, and 24 h after CLP/sham surgery and replaced by 0.6 mL of saline solution. To investigate the therapeutic effects, neostigmine, physostigmine, or placebo was administered by intraperitoneal injection four times over 24 h. The animals were evaluated regularly (clinical appearance score, breathing rate). All surviving animals were euthanized after 24 h by decapitation, and their organs were dissected and isolated for histopathologic examination. Blood was collected for biochemical analyses according to the time points in Figure 1. No antibiotics were administered in this model.

### 2.3. Anesthesia and Surgery

After intraperitoneal injection of 150 *μ*g/kg of medetomidine, 2 mg/kg of midazolam, and 5 *μ*g/kg of fentanyl, the sham operation (only laparotomy, no ligation or puncture of the cecum) was carried out or CLP was performed. CLP was performed as described in detail in the previous work [21, 22]. The vena jugularis interna was exposed and cannulated using a 26-gauge needle. A 3 Fr polyurethane catheter was inserted into the vein and tunneled under the coat to the back of the animal to allow easy blood sampling without stressing the animals or further anesthesia.

### 2.4. Weight and Clinical Appearance Score

The animals' weights were determined at *t* = 0, 6, 15, 20, and 24 h, using a laboratory precision balance (BP 221 S, Sartorius, Göttingen, Germany). A laboratory assistant blinded to the animals' group assignments assessed and graded the clinical appearance of the animals on a four-point scale ranging from 0 to 3. Three parameters were evaluated: eyes (0 = open; 1 = half open; 2 = crusting exudate; and 3 = closed, crusted, ensanguined), coat (0 = normal, shiny; 1 = dull; 2 = scrubby; and 3 = very scrubby, crusted), and movement (0 = eat, move, drink, and keep themselves clean; 1 = less movement; 2 = lethargic, movement only by contact; and 3 = no movement, lack of pain) [23].

### 2.5. Blood Sampling

#### 2.5.1. Blood Gas Analysis

Venous blood samples were collected at the five time points indicated in Figure 1. pH, pCO_2_, pO_2_, base excess, K^+^, Na^+^, Ca^2+^, Cl^−^, Hb, CO-Hb, Met-Hb, hematocrit, glucose, lactate, and SO_2_ were analyzed by a blood gas analyzer (IL GEM 3000, Instrumentation Laboratories, Kirchheim, Germany).

#### 2.5.2. Oxidative Burst Assay and Immunostaining with Anti-CD11b or Anti-CD62l

*(1) Oxidative Burst Assay*. The oxidative burst assay has been performed as described previously [24, 25]. For quantifying the oxidative burst, 2 × 10 *μ*L heparinized whole blood was collected at the five time points indicated in Figure 1. Both samples for each time point were divided into two groups (ROS sample group: without further in vitro stimulation and n-formyl-methionyl-leucyl phenylalanine (fMLP) sample group: with further fMLP in vitro stimulation). Each sample was suspended in 1 mL PBS (Dulbecco's without Ca and Mg, PAA, Pasching, Austria) and loaded with the fluorogenic substrates dihydrorhodamine (DHR; 10 *μ*L, 100 *μ*M) and carboxy-seminaphthorhodafluor-1-acetoxymethylester (SNARF/AM, 10 *μ*L, 10 *μ*M) (both purchased from Molecular Probes, Eugene, OR, USA). Following incubation for 10 min at 37°C, 10 *μ*L, 1 *μ*M fMLP (Sigma-Aldrich, Steinheim, Germany) was added to the fMLP sample group, not to the ROS sample group. Finally, after further incubation (30 min, 37°C), dead cells were counterstained with 10 *μ*L, 1.5 *μ*M propidium iodide (PI, Serva, Heidelberg, Germany). Subsequently, the samples were placed on ice to stop any further reaction.

*(2) Immunostaining with Anti-CD11b or Anti-CD62l*. For immunostaining, 10 *μ*L heparinized whole blood was collected at the five time points indicated in Figure 1. Samples were loaded with a 10 *μ*L aliquot of fluorescein isothiocyanate-conjugated (FITC) anti-rat CD62l-specific monoclonal antibody (Beckman Coulter GmbH, Krefeld, Germany) or a 10 *μ*L aliquot of FITC anti-rat CD11b-specific monoclonal antibody (BioLegend, San Diego, California, USA). After incubation (30 min), the samples were placed on ice.

*(3) Red Blood Cell Lysis and Flow Cytometric Measurement of Oxidative Burst and Adhesion Molecules*. Afterwards, 2 mL (4°C) red blood cell lysis buffer (8.3 g/L ammonium chloride, 1.68 g/L sodium bicarbonate, and 0.358 g/L EDTA; all Merck, Darmstadt, Germany) was added. Following 15 min incubation in the dark (4°C), 2 mL PBS was added to stop lysis. After a final washing step, the cells were dissolved in 200 *μ*L PBS.

For analysis, 5000 cells of each stained sample were acquired at 488 nm excitation (argon ion laser) using a FACSCalibur™ flow cytometer and the CellQuest Pro™ software (Becton Dickinson, San José, California, USA). The cells were analyzed immediately after preparation, and the tubes were kept on ice during data collection.

The oxidative burst was measured by the indicator dye DHR. The nonfluorescent DHR was oxidized intracellulary to green fluorescent rhodamine 123. The amount of rhodamine 123 was proportional to generated ROS. The dead cells were identified by increased PI fluorescence (emission above 600 nm) and lack of esterase activity determined based on SNARF1-related orange fluorescence. SNARF1/AM (nonfluorescent) was cleaved in vital leukocytes by esterases to SNARF1. FITC-labeled antibody produced fluorescence, allowing the quantification of expression of CD62l or CD11b. PMNs were identified by their typical side and forward scatter pattern and their SNARF-1 fluorescence. Evaluation and measurement of each specimen have been conducted in double measurement determination. The results of cellular fluorescence were expressed as molecules of equivalent soluble fluorochrome (MESF).

#### 2.5.3. Analysis of AChE and BChE Activity

For the AChE or BChE activity analysis, 10 *μ*L of whole blood was collected at the five time points indicated in Figure 1. We used ChE Check (Securetec Detektions-Systeme AG, Neubiberg, Germany; In Vitro Diagnostics Guideline 98/79/EG; DIN EN ISO 18113-2 and -3), a validated point-of-care testing device, to determine the AChE and BChE activities according to the instructions from the manufacturer. This enzymatic assay enables the rapid and precise determination of AChE and BChE activities in whole blood without sample pretreatment. The AChE and BChE activities were assayed at room temperature using two separate test kits for each enzyme by indirectly measuring the production of thiocholine from the hydrolysis of their respective specific substrates, acetylthiocholine iodide and s-butyrylthiocholine iodide. Thiocholine further reacts with 5,5′-dithio-bis-2-nitrobenzoic acid (DTNB, Ellman's reagent) as a chromogenic reagent to produce a yellow product, the 5-thio-2-nitrobenzoate anion (TNB, Ellman's anion). The production of TNB was monitored at 470 nm. Enzymatic activities were expressed as U/L for BChE and as U/gHb for AChE according to the manufacturer's instructions [26].

### 2.6. Histopathologic Examination

The lungs, livers, kidneys, and spleens of the animals were dissected from the surrounding tissue. Organs were prepared for blinded histopathologic assessment. Tissues were fixed in 4% neutral-buffered formalin for 48 h. After embedding in paraffin, the tissues were sectioned with a microtome at 4 *μ*m and stained with hematoxylin-eosin. The slides were examined under conventional light microscopy (Leitz DM-RBE, Leica, Wetzlar, Germany). A pathologist blinded to the study groups assessed and scored the degree of tissue injuries using a scoring system developed by Akinci et al. with a four-point scale ranging from 0 to 3. The total organ injury score was calculated by adding all parameters for lungs, livers, kidneys, and spleens (maximum score of 45) [11].

### 2.7. Statistical Analysis

G∗Power 3.1.3 software (University Düsseldorf, Germany) was used to determine the sample size (power 80%, significance level alpha = 5%). All data in the text, tables, and figures are displayed as the means ± standard error of the mean (SEM) or standard deviation (SD). For the statistical analysis, we applied the Kolmogorov-Smirnov test to confirm the normal distribution for each group. Statistical significance was tested using analysis of variance, Friedman's two-way ANOVA, or the Kruskal-Wallis test. Post hoc analysis was performed by using the Bonferroni comparison test. *p* < 0.05 was considered statistically significant. The statistical software used to conduct the analyses was SPSS 24 (SPSS Inc., Chicago, IL, USA).

## 3. Results

### 3.1. Survival Time

**Figure 2:**
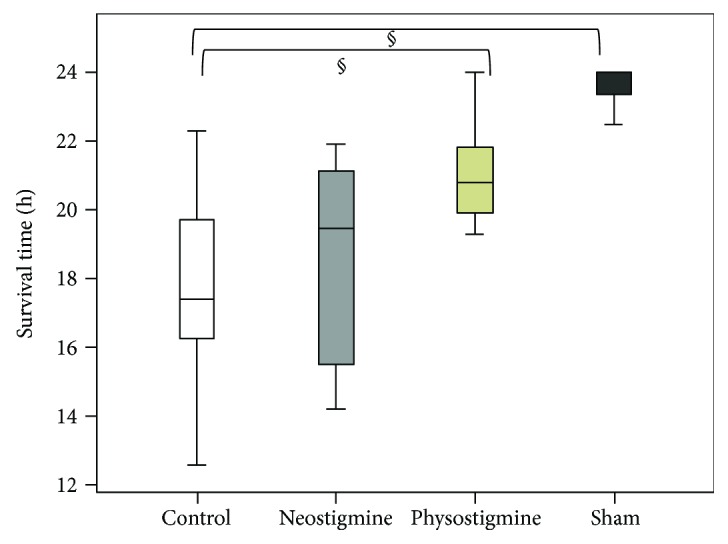
Illustration of the mean survival times of the animals in the four groups. Immediately after CLP induction, the rats were treated with either physostigmine, neostigmine, or saline four times over 24 h. While physostigmine-treated animals survived significantly longer than the control rats receiving the saline, the mean survival time of neostigmine-treated animals was not significantly longer (mean survival time in hours, control (*n* = 18): 17.5 ± 2.6 vs. physostigmine (*n* = 14): 20.5 ± 2.3 (^§^*p* < 0.05); control vs. neostigmine (*n* = 14): 18.3 ± 2.9 (*p* = n.s.)). Box plots in panels represent medians with 25% and 75% percentiles; error bars are minimum and maximum values, Kruskal-Wallis test followed by Bonferroni's pairwise comparison test.

Immediately after CLP induction, the rats were treated with either physostigmine, neostigmine, or saline four times over 24 h. Physostigmine-treated animals survived significantly longer than the control rats receiving the saline (mean survival time in hours, control: 17.5 ± 2.6 vs. physostigmine: 20.5 ± 2.3 (*p* < 0.05); control vs. neostigmine 18.3 ± 2.9 (n.s.), Figure 2).

### 3.2. Blood Gas Analysis, Clinical Score, and Weight

Table 1 shows the weights and the clinical appearance scores of the animals at various treatment times. No significant difference in the breathing rates was observed between the groups during the entire experiment (*p* = n.s.). Body weight did not change over time in any group (*p* = n.s.).

The animals appeared healthy for approximately 10 h. Then, the animals in the control and treatment groups became subsequently ill and exhibited the typical behavior and appearance described by Wichtermann et al. [23]. The clinical appearance scores for eyes, coat, and movement were significantly higher in the placebo and treatment groups than the sham group (^$^*p* < 0.05, Table 1). pCO_2_, pO_2_, K^+^, Na^+^, Ca^2+^, Cl^−^, Hb, CO-Hb, Met-Hb, hematocrit, and SO_2_ did not change significantly during the entire experiment in any group (*p* = n.s., Table 2). The treated animals and the control group showed increasing lactate levels during the experiment, which reached significance at *t* ≥ 20 h (^∗^*p* < 0.05 versus *t* = 0, Table 2). In the control group, base excess was significantly reduced at *t* ≥ 20 h (^∗^*p* < 0.05 versus *t* = 0, Table 2), but not in the treated animals or the sham group. After 15 h, the pH and base excess of the sham group were significantly higher than those of the control and treatment groups (^$^*p* < 0.05 versus control/treatment, Table 2). Glucose levels significantly decreased over time in all groups (^∗^*p* < 0.05 versus *t* = 0). At *t* ≥ 15 h, glucose levels were significantly higher in the sham group than in the control and treatment groups (^$^*p* < 0.05 versus the control/treatment group).

### 3.3. Production of Reactive Oxygen Species by PMN

**Figure 3:**
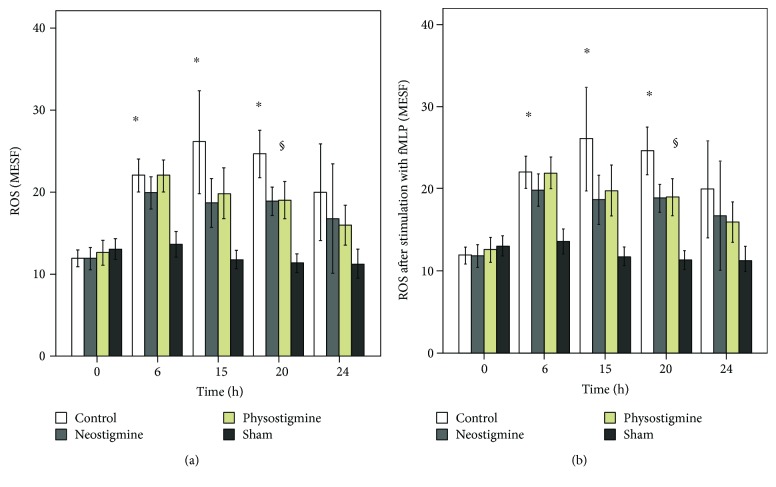
(a, b) Effects of physostigmine and neostigmine treatment on ROS production of PMNs during CLP-induced sepsis in rats. (a) ROS production in the sham group (*n* = 14) did not change significantly during the entire experiment (*p* = n.s.). In the control (*n* = 18), physostigmine (*n* = 14) and neostigmine (*n* = 14) groups' ROS production increased significantly at *t* = 6, 15, and 20 h, respectively (^∗^*p* < 0.05). (b) ROS production by PMNs following in vitro stimulation with fMLP increased significantly at *t* = 6, 15, and 20 h in the control group (^∗^*p* < 0.05). The animals treated with either physostigmine or neostigmine showed decreased levels of ROS production, which reached significance at *t* = 20 h (^§^*p* < 0.05). The comparison between the animals treated with either physostigmine or neostigmine revealed no significant differences in ROS production over the course of the experiment. The data are presented as the mean ± SEM. ^∗^*p* < 0.05 versus *t* = 0; ^§^*p* < 0.05 versus the control, analysis of variance followed by Bonferroni's multiple comparisons test.

Figures 3(a) and 3(b) show that ROS production in the sham group (*n* = 14) did not change significantly during the entire experiment. CLP-induced sepsis led to a strong increase in ROS production, which showed significance at *t* = 6, 15, and 20 h in the control (*n* = 18), physostigmine (*n* = 14), and neostigmine (*n* = 14) groups (^∗^*p* < 0.05, Figure 3(a)). In addition, the ROS production by PMNs following in vitro stimulation with fMLP increased significantly at *t* = 6, 15, and 20 h in the control group (^∗^*p* < 0.05, Figure 3(b)). In contrast, the animals treated with either physostigmine or neostigmine showed significantly decreased levels of ROS production at *t* = 20 h compared to those in the control group (^§^*p* < 0.05). The comparison between animals treated with physostigmine or neostigmine showed no significant differences in ROS production over the whole course of the experiment (*p* = n.s.).

### 3.4. Expression of CD11b and CD62l on the Surface of PMNs

**Figure 4:**
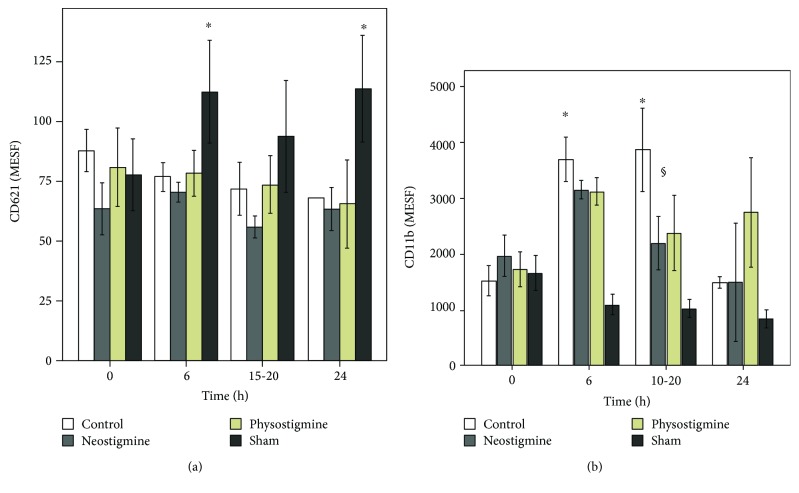
(a, b) Effects of physostigmine and neostigmine treatment on CD11b and CD62l expression levels of PMNs during CLP-induced sepsis in rats. (a) In the sham group (*n* = 14), CD62l expression was significantly higher 6 h after beginning the experiment (^∗^*p* < 0.05). In the control (*n* = 18) and treatment groups (*n* = 14 neostigmine, *n* = 14 physostigmine), no significant differences in CD62l expression were observed over the course of the experiment. (b) CD11b expression in the sham group (*n* = 14) did not change significantly during the entire experiment (*p* = n.s.). CLP-induced sepsis led to a strong increase in CD11b expression, which reached significance at *t* = 6 and 15-20 h in the control group (*n* = 18, ^∗^*p* < 0.05). In the physostigmine (*n* = 14) and neostigmine (*n* = 14) groups, CD11b expression increased, reaching significance at *t* = 6 h. At *t* = 15-20 h, CD11b expression was significantly reduced in the animals treated with physostigmine or neostigmine compared with the control group (^§^*p* < 0.05). The data are presented as the mean ± SEM. ^∗^*p* < 0.05 versus *t* = 0; ^§^*p* < 0.05 versus the control, Friedman's two-way ANOVA and Kruskal-Wallis test followed by Bonferroni's pairwise comparison test.

In the sham group, CD62l was significantly higher 6 h after beginning the experiment (Figure 4(a)). In the control and treatment groups, no significant differences were observed in CD62l expression over the whole course of the experiment.

CD11b expression in the sham group did not change significantly during the entire experiment (*p* = n.s., Figure 4(b)). CLP-induced sepsis led to a strong increase in CD11b expression, which showed significance at *t* = 6 and 15-20 h in the control group (^∗^*p* < 0.05). In the physostigmine and neostigmine groups, CD11b expression increased, showing significance at *t* = 6 h (^∗^*p* < 0.05). At *t* = 15-20 h, CD11b expression was significantly reduced in the animals treated with physostigmine or neostigmine compared with the control group (^§^*p* < 0.05).

### 3.5. Histopathologic Examination

**Figure 5:**
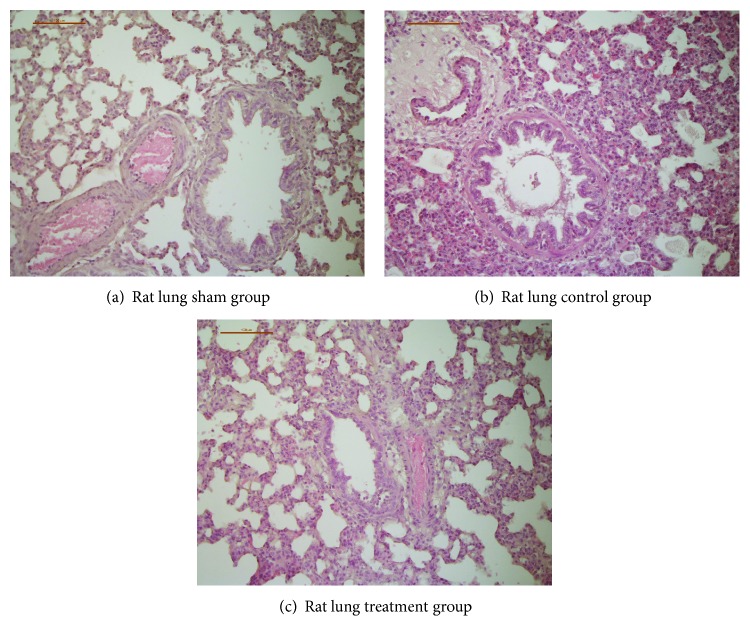
(a, b, and c) Histopathologic images of rat lungs. CLP induced an increase in organ injury in all examined tissues (median histological injury score in the control group 14.0 ± 2.7 vs. the sham group 7.7 ± 2.5, *p* < 0.05, Kruskal-Wallis test followed by Bonferroni's pairwise comparison test). The median histological score of neostigmine-treated or physostigmine-treated animals was not significantly different compared to the control group animals (*p* = n.s. vs. the control group). We present examples of histopathologic images of rat lungs, since the differences between the groups are most apparent compared to other examined tissues: (a) Rat lung of the sham group with little to no blood stasis and small vessel lumina. No signs of perivascular or interstitial inflammation, lung injury score 1. (b) Rat lung of the control group with marked alveolar hemorrhage, congestion, and dilated, hyperemic blood vessels. PMN infiltrates lead to a strong perivascular and interstitial inflammation, lung injury score 7. (c) Rat lung of an animal treated with physostigmine with intra-alveolar edema and congestion. Only mild PMN infiltrates and little alveolar hemorrhage, lung injury score 4.

Histological examination of the organs of the sham group showed a median histological injury score of 7.7 ± 2.5. The control group showed increased organ injury in the lungs, livers, kidneys, and spleens with a median histological injury score of 14.0 ± 2.7 (*p* < 0.05 vs. the control group). Neostigmine-treated animals showed a median histological score of 11.3 ± 1.3, and physostigmine-treated animals showed a median score of 12.7 ± 3.6 (*p* = n.s. vs. the control group). Examples of histopathologic images of rat lungs are shown in Figures 5(a)–5(c).

### 3.6. Relative AChE and Relative BChE Activity during CLP

**Figure 6:**
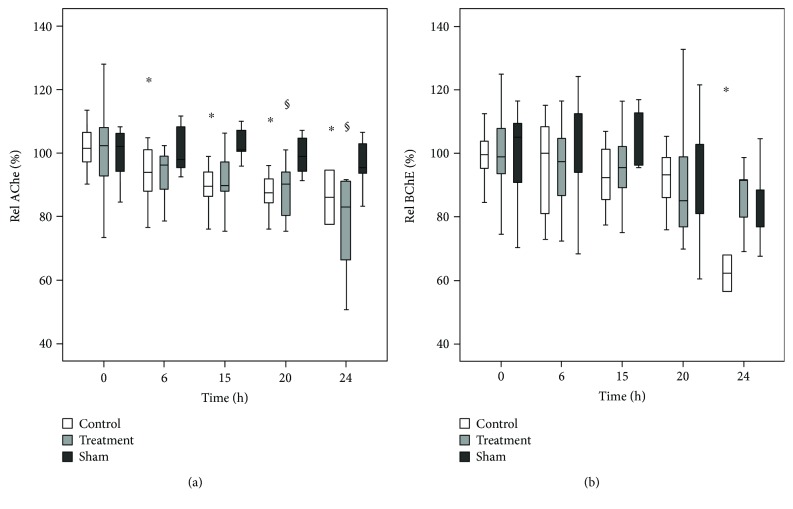
(a, b) Relative AChE and relative BChE activity during CLP-induced sepsis in rats (results for physostigmine- and neostigmine-treated animals are combined as “treatment group”). Relative activity for the given time point was calculated as the ratio between the measured absolute enzyme activity for the given time point and the enzyme activity at *t* = 0. (a) In the control group (*n* = 18), AChE activity decreased versus baseline, being significant at *t* = 6, 15, 20, and 24 h (^∗^*p* < 0.05). The sham group (*n* = 14) did not show any differences in AChE activity during the observation time of 24 h. In the treatment groups (physostigmine and neostigmine, *n* = 28), AChE activity significantly decreased at *t* = 20 and 24 h (^§^*p* < 0.05). (b) There were no significant differences of BChE activity until *t* = 20 h in any group. At *t* = 24 h, BChE activity was significantly decreased in the control group (*n* = 18, ^∗^*p* < 0.05). Box plots in panels represent medians with 25% and 75% percentiles; error bars are minimum and maximum values. ^∗^*p* < 0.05 versus *t* = 0 in the placebo group. ^§^*p* < 0.05 versus *t* = 0 in the treatment group, Friedman's two-way ANOVA followed by Bonferroni's pairwise comparison test.

Figures 6(a) and 6(b) show the relative AChE and relative BChE activities over time. The relative activity at a given time point was calculated as the ratio between the measured absolute enzyme activity at the given time point and the enzyme activity at *t* = 0. In the control group, AChE activity decreased compared to that at baseline, showing significance at *t* = 6, 15, 20, and 24 h. In the sham group, no differences in AChE activity were observed throughout the observation period of 24 h. In the treatment group, AChE activity significantly decreased at *t* = 20 and 24 h (Figure 6(a)). No significant differences in BChE activity were observed until *t* = 20 h in any group. At *t* = 24 h, we observed a decreased BChE activity in the control group (Figure 6(b)).

## 4. Discussion

The primary objective of the present investigation was to evaluate the potential therapeutic effects of cholinesterase inhibitors on PMN functions during the early phase of sepsis and to investigate the roles of AChE and BChE as inflammatory markers in CLP-induced sepsis.

### 4.1. PMN Functions during CLP-Induced Sepsis

CLP causes lethal peritonitis by polymicrobial infection and has been identified as a relevant animal model for human sepsis [1]. CLP in rats causes an early septic period with hyperdynamic circulation and hyperglycemia, while the late septic period shows low levels of serum glucose and high levels of serum lactate [14]. These observations were reproduced in the present study (Table 2). Following CLP, most rats followed a predictable pattern of behavior and appearance, which was previously described by Wichterman et al. [23]. Initially, the rats rapidly recovered from anesthesia and generally appeared healthy for approximately 10 h. The animals in control and treatment groups became subsequently ill and showed higher clinical appearance scores compared to the animals in the sham group (Table 1).

The present results clearly demonstrate a significant, time-dependent increase in ROS production by PMNs after CLP induction (Figures 3(a) and 3(b)). ROS production peaked at *t* = 15 h and then decreased until *t* = 24 h. The course of ROS production indicates an early, hyperinflammatory and a late, hypoinflammatory phase of sepsis. In the literature, CLP has been shown to activate the peripheral innate immune system, leading to the release of many inflammatory mediators such as ROS [1]. Our results further show a significant downregulation of CD62l expression in septic animals compared to the sham group, which is consistent with the previous studies. Thiel et al. showed that in critically ill patients with septic shock, the level of L-selectin expression was decreased in circulating PMNs [27]. The present results demonstrate a significant upregulation of CD11b expression on the PMN surface in the control animals. Increased expression of CD11b is part of the systemic inflammatory response syndrome during the early phase of sepsis [28]. Leukocyte recruitment is critical for the effective elimination of invading pathogens. However, excessive leukocyte accumulation during inflammation mediated by the overexpression of adhesion molecules can lead to tissue damage. In contrast, insufficient cell activation and subsequent impaired immune cell trafficking can result in host immunodeficiency [9]. In the present study, CLP-induced sepsis increased interstitial inflammation and histopathological organ injury in general terms. While the organs of the sham group showed a median histological injury score of 7.7 ± 2.5, the control group showed significantly increased organ injury with a median histological injury score of 14.0 ± 2.7 (maximum score of 45). Therefore, the regulation of leukocyte recruitment must be controlled precisely since it plays a pivotal role in the clinical development and manifestation of sepsis or septic shock. Further clarification of the role of cholinesterase inhibitors in the therapeutic manipulation of PMN actions in the early phase of sepsis is imperative.

### 4.2. Effects of Neostigmine or Physostigmine Treatment on PMN Functions

The aim of the present study was to investigate the effects of peripherally and centrally acting cholinesterase inhibitors on PMN functions during CLP-induced sepsis in rats. Previous studies have shown that the CAP can be pharmacologically activated by cholinesterase inhibitors since elevated levels of acetylcholine inhibit the synthesis of proinflammatory cytokines [5–8, 29]. However, no data are available regarding the in vivo effects of the CAP on PMN functions during the early phase of sepsis. Compared to Hofer et al., we did not use ketamine as an anesthetic for CLP induction and avoided further anesthetics for blood sampling during the experiment to mitigate potential pharmacologic side effects. Ketamine is a noncompetitive inhibitor of the nicotinic acetylcholine receptor that may cause the receptor to be unresponsive to acetylcholine elevations induced by cholinesterase inhibitors [10].

Immediately after CLP induction, rats were treated with either physostigmine, neostigmine, or saline four times over 24 h. Physostigmine and neostigmine are the two main cholinesterase inhibitors available in clinical practice, which are used in the treatment of colonic ileus and central anticholinergic syndrome or as an adjuvant in pain treatment [30, 31]. One difference between neostigmine and physostigmine is the site of action. We compared treatment with physostigmine, which is lipid-soluble and crosses the blood-brain barrier, to treatment with neostigmine, which only acts in the periphery [12, 25].

The results of the present study demonstrate that physostigmine and neostigmine have protective effects on PMN functions during CLP-induced sepsis. We found a significant reduction in ROS production by treatment with physostigmine as well as neostigmine. ROS production plays a central role in modulating mortality in experimental and clinical sepsis, and residual levels of ROS are probably necessary for the clearance of bacterial infections. The observed downregulation of ROS production upon cholinesterase inhibition in the early phase of sepsis seems to be important in the further course of sepsis and is potentially an underlying reason for the improved outcomes among the treated animals, which showed less lactate acidosis and extended survival times (Figure 2).

Furthermore, the present study demonstrated a significant inhibitory effect of physostigmine or neostigmine on the expression of CD11b on the surface of PMNs. Peter et al. showed that the number of rolling leukocytes as well as leukocyte-endothelial interactions was significantly reduced by the application of physostigmine during experimental endotoxemia [9]. On the basis of these observations, the reduced leukocyte-endothelial interactions seemed to be due to reduced activation of adhesion molecules. Histopathologic examination of tissue injury after 24 h observation time showed reduced injury scores by trend in physostigmine- and neostigmine-treated animals, which were not significantly different from that of the control group.

Administration of physostigmine or neostigmine reduced ROS production and CD11b upregulation in the early phase of sepsis, which was probably attributable to the peripheral effects of acetylcholine on the alpha7-nicotinic acetylcholine receptor expressed on PMNs. This gives rise to the hypothesis that the CAP modulates the inflammatory functions of PMNs and their respective interactions by integrating independent cell-specific pathways [9].

The animals treated with physostigmine showed significantly improved survival times compared with those of the control animals. In neostigmine-treated animals, in contrast, survival times were not significantly improved. Therefore, peripheral stimulation of cholinergic receptors is sufficient to confer protective effects on PMN functions, but for improved survival times during the early phase of sepsis the additional lipid-soluble component of physostigmine seems to be required.

These findings are consistent with those of the previous study by Kox et al. [12], who reported no protective effects of neostigmine on ventilation-induced lung injury. Akinci et al. investigated the effects of neostigmine on organ injury in mice during endotoxemia and failed to demonstrate protective effects of neostigmine [11]. The authors suspected that the reason for the higher mortality rates was the clinically important cardiovascular side effects of neostigmine, such as bradycardia. We used neostigmine in a dose of 75 *μ*g/kg and physostigmine in a dose of 100 *μ*g/kg, which are both comparable with the doses used in the literature [10, 12, 32].

Interestingly, Hofer et al. showed improved survival in murine CLP-induced sepsis after the application of neostigmine as well as physostigmine [10]. One main difference is the varying times of observation. Since the focus of the present study was on the early phase of sepsis, our observation time stopped after 24 hours. In contrast, Hofer et al. observed the animals for seven days. The integrity of the blood-brain barrier may be compromised during sepsis and by drugs, including neostigmine, whose action is peripherally restricted under normal conditions may reach the brain and act centrally. This is in accordance with our present findings that some protective effects on PMN functions are obtained at the later 20 h time point, when treatment with neostigmine may theoretically affect the brain.

### 4.3. The Role of AChE and BChE as Inflammatory Markers in CLP

Recent studies have shown that AChE and BChE serve as diagnostic markers of low-grade systemic inflammation [13–15]. Rapid changes in cholinesterase activity have also been reported in patients after acute trauma, infections, burns, and critical illness [16–20]. However, due to high variability in the onset, etiology, and progress of clinical conditions, it was difficult to determine whether the change in the enzyme activity correlated with the emergence of disease or is affected by concomitant factors such as substance abuse, liver disease, or nephrotic disease [19]. This is the first study to investigate the roles of AChE and BChE as inflammatory markers under standardized experimental conditions.

The findings of our study show that CLP-induced sepsis caused a significant and time-dependent decline in AChE activity (Figure 6(a)). In the control group, AChE activity decreased compared to that at baseline, showing significance at *t* = 6, 15, 20, and 24 h. At *t* = 24 h, AChE activity was significantly reduced by approximately 19%. Decreased AChE activity can be associated with elevated proinflammatory markers (ROS production, CD11b expression) in the circulation during CLP-induced sepsis. Therefore, the AChE decrease observed in our study is likely related to the pathophysiological response to CLP, although the underlying mechanism remains unclear. In a study on burn patients, the decrease in cholinesterase activity has been suggested to have multifactorial causes, such as decreased liver synthesis, capillary leakage, or high enzyme consumption due to stress-induced catabolic metabolism [20]. The decrease in AChE activity correlated with the development of a lactate acidosis, a conventional indicator of poor outcomes in sepsis. Compared to lactate acidosis or other conventional laboratory tests that are used to diagnose inflammation [18, 33, 34], AChE activity changes can be detected much earlier (at *t* = 6 h). Therefore, AChE may play an important role in identifying early systemic inflammation.

In the treated animals, AChE activity also decreased compared to that at baseline, showing significance at *t* = 20 and 24 h. The delayed decrease in AChE activity in the treated animals compared to that in the control group could be associated with the positively influenced course of sepsis in the treated animals. Therefore, AChE activity may also play an important role in estimating the severity of inflammatory diseases.

In contrast, BChE activity did not change in any group until *t* = 20 h (Figure 6(b)). At *t* = 24 h, we observed reduced activity in the control group, but not in the treatment or sham groups. The necessity and precise physiological function of BChE remain largely unknown [16, 26]. BChE is synthesized in the liver [17] and has therefore been conventionally used as a liver function biomarker. Indeed, the work of al-Kassab and Vijayakumar suggested the importance of BChE as an indicator of hepatic dysfunction in septic syndrome [14]. In the present study, we showed a decrease in BChE activity at 24 h, which could be associated with hepatic dysfunction in septic rats. This is in accordance with the observed hypoglycemia in septic animals (Table 2) compared to the sham group, which may also be a sign of hepatic dysfunction.

The CLP model used in this study has been shown to be a suitable model for evaluating AChE and BChE as inflammatory markers under standardized experimental conditions. Our data indicate that BChE activity can be regarded as a marker with delayed decrease, while AChE activity is an acute marker and may serve as an early indicator of acute systemic inflammation [35].

## 5. Limitations

The animals were not monitored hemodynamically in our study, since we wanted to avoid additional manipulation of the animals or any further anesthesia.

Further inflammatory biomarker analyses (e.g., CRP and IL-6), which would require additional blood samples in small animals, were not included.

## 6. Conclusion

While AChE activity could serve as an acute marker of systemic inflammation in CLP-induced sepsis, BChE activity shows a delayed decrease, which is consistent with the previous studies that demonstrated the remarkable predictive value of cholinesterases for mortality in critically ill patient populations. Our results suggest that the administration of physostigmine or neostigmine after CLP induction in rats leads to a significant reduction in ROS production and CD11b upregulation during the early phase of sepsis. Centrally acting physostigmine, but not peripherally acting neostigmine, improved survival times significantly. These findings provide a rationale for further exploration and may be of interest in clinical practice. Anticholium® per Se, for example, is a currently ongoing placebo-controlled trial [36] exploring the adjunctive use of physostigmine in patients with perioperative sepsis and septic shock.

## Figures and Tables

**Table 1:** Effects of various treatments on the clinical appearance score, breathing rate, and body weights during the observation period (results for physostigmine- and neostigmine-treated animals are combined as “treatment group”).

Clinical score	Time (hours)	Control (mean ± SD)	Treatment (mean ± SD)	Sham (mean ± SD)
Breathing rate (per min)	**0**	106 ± 7.6 (*n* = 18)	108 ± 11.8 (*n* = 28)	105 ± 19.3 (*n* = 14)
	**15**	107 ± 19.6 (*n* = 15)	114 ± 13.7 (*n* = 27)	120 ± 11.4 (*n* = 14)
	**20**	90 ± 22.3 (*n* = 9)	120 ± 17.3 (*n* = 16)	115 ± 18.0 (*n* = 14)
	**24**	90 ± 14.1 (*n* = 3)	115 ± 26.6 (*n* = 9)	126 ± 12.8 (*n* = 12)

Coat (0-3)	**0**	0 ± 0 (*n* = 18)	0 ± 0 (*n* = 28)	0 ± 0 (*n* = 14)
	**15**	2.2 ± 0.6^∗^^,$^ (*n* = 15)	2.2 ± 0.5^∗^^,$^ (*n* = 27)	0.45 ± 0.3 (*n* = 14)
	**20**	2.5 ± 0.7^∗^^,$^ (*n* = 9)	2.7 ± 0.5^∗^^,$^ (*n* = 16)	0.36 ± 0.7 (*n* = 14)
	**24**	3.0 ± 0.0^∗^^,$^ (*n* = 3)	2.7 ± 0.5^∗^^,$^ (*n* = 9)	0.29 ± 0.5 (*n* = 12)

Movement (0-3)	**0**	0 ± 0 (*n* = 18)	0 ± 0 (*n* = 28)	0 ± 0 (*n* = 14)
	**15**	1.7 ± 0.6^∗^^,$^ (*n* = 15)	1.8 ± 0.6^∗^^,$^ (*n* = 27)	0.25 ± 0.3 (*n* = 14)
	**20**	2.5 ± 0.5^∗^^,$^ (*n* = 9)	1.9 ± 0.8^∗^^,$^ (*n* = 16)	0.1 ± 0.3 (*n* = 14)
	**24**	3.0 ± 0.0^∗^^,$^ (*n* = 3)	2.1 ± 0.7^∗^^,$^ (*n* = 9)	0.14 ± 0.4 (*n* = 12)

Eyes (0-3)	**0**	0 ± 0 (*n* = 18)	0 ± 0 (*n* = 28)	0 ± 0 (*n* = 14)
	**15**	2.1 ± 0.3^∗^^,$^ (*n* = 15)	2.2 ± 0.6^∗^^,$^ (*n* = 27)	0.4 ± 0.7 (*n* = 14)
	**20**	2.4 ± 0.5^∗^^,$^ (*n* = 9)	2.7 ± 0.5^∗^^,$^ (*n* = 16)	0.2 ± 0.4 (*n* = 14)
	**24**	2.5 ± 0.7^∗^^,$^ (*n* = 3)	2.7 ± 0.5^∗^^,$^ (*n* = 9)	0.3 ± 0.5 (*n* = 12)

Weight (g)	**0**	250 ± 31.9 (*n* = 18)	265 ± 40.6 (*n* = 28)	248 ± 23.9 (*n* = 14)
	**15**	247 ± 43.4 (*n* = 15)	256 ± 73.5 (*n* = 27)	256 ± 23.3 (*n* = 14)
	**20**	264 ± 35.6 (*n* = 9)	262 ± 56.6 (*n* = 16)	259 ± 22.9 (*n* = 14)
	**24**	246 ± 44.6 (*n* = 3)	282 ± 71.6 (*n* = 9)	270 ± 20.9 (*n* = 12)

There was no significant difference of the breathing rates during the entire experiment in any group (*p* = n.s.). The body weights did not change over time in any group (*p* = n.s.). The clinical appearance scores for eyes, coat, and movement were significantly higher in the control and treatment group than in the sham group (^$^*p* < 0.05 vs. the sham group). At *t* = 15 and 24 h, the clinical appearance score was in the placebo group and treatment groups significantly higher than at *t* = 0 (^∗^*p* < 0.05 vs. *t* = 0). The data are displayed as the means ± standard deviation (SD). ^∗^*p* < 0.05 versus *t* = 0. ^$^*p* < 0.05 versus the sham group.

**Table 2:** Results of blood gas analysis during the observation period of 24 h (results for physostigmine- and neostigmine-treated animals are combined as “treatment group”).

Blood gas analysis	Time (h)	Control (mean ± SD)	Treatment (mean ± SD)	Sham (mean ± SD)
pH	**0**	7.37 ± 0.03 (*n* = 18)	7.37 ± 0.03 (*n* = 28)	7.37 ± 0.04 (*n* = 14)
	**15**	7.46 ± 0.07^∗^ (*n* = 15)	7.47 ± 0.05^∗^ (*n* = 27)	7.52 ± 0.02^§,^^∗^ (*n* = 14)
	**20**	7.37 ± 0.09 (*n* = 9)	7.44 ± 0.12 (*n* = 13)	7.48 ± 0.07 (*n* = 10)
	**24**	7.35 ± 0.11 (*n* = 3)	7.41 ± 0.15 (*n* = 9)	7.46 ± 0.09^§^ (*n* = 12)

Base excess (mmol/L)	**0**	2.9 ± 1.38 (*n* = 18)	2.74 ± 1.64 (*n* = 28)	2.65 ± 1.64 (*n* = 14)
	**15**	3.0 ± 3.58 (*n* = 15)	2.91 ± 4.30 (*n* = 27)	8.59 ± 5.39^§^ (*n* = 14)
	**20**	-0.23 ± 5.46 (*n* = 9)	2.22 ± 7.32 (*n* = 13)	7.19 ± 4.89^§^ (*n* = 10)
	**24**	-2.66 ± 6.66^∗^ (*n* = 3)	-0.69 ± 9.19 (*n* = 9)	6.38 ± 5.16^§^ (*n* = 12)

Lactate (mmol/L)	**0**	1.36 ± 0.41 (*n* = 18)	1.37 ± 0.41 (*n* = 28)	1.28 ± 0.29 (*n* = 14)
	**15**	1.64 ± 0.54 (*n* = 15)	1.32 ± 0.50 (*n* = 27)	1.36 ± 0.49 (*n* = 14)
	**20**	6.59 ± 2.43^∗^ (*n* = 9)	4.66 ± 2.82^∗^ (*n* = 13)	1.47 ± 0.39 (*n* = 10)
	**24**	6.78 ± 3.67^∗^ (*n* = 2)	5.35 ± 4.61^∗^ (*n* = 19)	1.70 ± 1.11^§^ (*n* = 12)

Glucose (mg/dL)	**0**	326.68 ± 86.16 (*n* = 18)	325.7 ± 69.54 (*n* = 28)	352.50 ± 30.06 (*n* = 14)
	**15**	90.78 ± 13.41^∗^ (*n* = 15)	91.95 ± 21.84^∗^ (*n* = 27)	134.57 ± 11.70^§,^^∗^ (*n* = 14)
	**20**	41.75 ± 23.28^∗^ (*n* = 9)	68.66 ± 29.37^∗^ (*n* = 13)	148.45 ± 17.80^§,^^∗^ (*n* = 10)
	**24**	29.92 ± 34.38^∗^ (*n* = 2)	65.56 ± 47.27^∗^ (*n* = 9)	142.18 ± 32.15^§,^^∗^ (*n* = 12)

pCO_2_, pO_2_, K^+^, Na^+^, Ca^2+^, Cl^−^, Hb, CO-Hb, Met-Hb, hematocrit, and SO_2_ did not change significantly during the entire experiment in any group (data not shown). The treated animals and the control group showed an increase of lactate levels over time, being significant at *t* ≥ 20 h (^∗^*p* < 0.05 versus *t* = 0). In the control group, base excess was significantly reduced at *t* ≥ 20 h (^∗^*p* < 0.05 versus *t* = 0), but not in the treated animals or the sham group. After 15 h, pH and base excess of the sham group were significantly higher than in the control and treatment groups (^§^*p* < 0.05 versus placebo/treatment). Glucose levels significantly decreased over time in all groups (^∗^*p* < 0.05 versus *t* = 0). At *t* ≥ 15 h, glucose levels were significantly higher in the sham group than in the control and treatment groups (^§^*p* < 0.05 versus control/treatment). The data are presented as the mean ± SD. ^∗^*p* < 0.05 versus *t* = 0; ^$^*p* < 0.05 versus the control.

## Data Availability

The data used to support the findings of this study are available from the corresponding author upon request.
